# Supplementary material for: Rapidly damping hydrogels engineered through molecular friction
Source: Nat Commun. 2024 Jun 8;15:4895. doi: 10.1038/s41467-024-49239-4 (PMC11162443; doi:10.1038/s41467-024-49239-4)
Supplement: Supplementary file 1 — Supplementary Information [file 41467_2024_49239_MOESM1_ESM.pdf]

Supplementary Information

Rapidly damping hydrogels engineered through molecular friction

Zhengyu Xu[†], Jiajun Lu[†], Di Lu, Yiran Li, Hai Lei, Bin Chen, Wenfei Li, Bin Xue[*], Yi Cao[*], Wei Wang[*]

***Correspondence to: wangwei@nju.edu.cn; caoyi@nju.edu.cn; xuebinnju@nju.edu.cn**

This PDF file includes:

Supplementary Methods
Supplementary Figures 1 to 31

Table of contents

Supplementary methods 4

Synthesis and reaction efficiency of CD-PEG-CD 4

Determination of PEG-CD in the hydrogel network 4

Swelling and water content measurements 5

Scanning electron microscopy (SEM) 5

Determination of the diffusion speed at zero pulling forces 5

Rheological Measurements 6

Supplementary Figures 7

Supplementary Figure 1 7

Supplementary Figure 2 7

Supplementary Figure 3 8

Supplementary Figure 4 8

Supplementary Figure 5 8

Supplementary Figure 6 9

Supplementary Figure 7 9

Supplementary Figure 8 10

Supplementary Figure 9 11

Supplementary Figure 10 11

Supplementary Figure 11 12

Supplementary Figure 12 12

Supplementary Figure 13 13

Supplementary Figure 14 14

Supplementary Figure 15 14

Supplementary Figure 16 15

Supplementary Figure 17 15

Supplementary Figure 18 16

Supplementary Figure 19 16

Supplementary Figure 20 17

Supplementary Figure 21 17

Supplementary Figure 22 18

Supplementary Figure 23 19

Supplementary Figure 24 19

Supplementary Figure 25 20

Supplementary Figure 26.. 21
Supplementary Figure 27.. 21
Supplementary Figure 28.. 22
Supplementary Figure 29.. 23
Supplementary Figure 30.. 24
Supplementary Figure 31.. 25

Supplementary methods

Synthesis and reaction efficiency of CD-PEG-CD

Typically, NHS-PEG-NHS (2 or 5 kDa) was dissolved in DMSO to a concentration of 10 mM. Then, the amino-modified CD (α-CD-NH_2 or β-CD-NH_2) was added to a concentration of 80 mM. The mixture was stirred overnight and dialyzed in ddH_2O for over 48 hours using a dialysis bag (500 Da). Finally, the products were lyophilized and stored at 4 °C. The reaction efficiency was determined using an ultraviolet visible (UV–vis) spectrophotometer (Jasco, V550) and methyl orange. Typically, the calibration curve of CD concentrations was first measured according to the decrease in UV absorbance at 557 nm since CD could form a complex with methyl orange and fade the colour of the solutions. Then, CD-PEG-CD solutions were mixed with methyl orange solutions, and the mass concentrations of CD (C_1) were determined according to the calibration curve. The reaction efficiency was calculated as: $\frac{C_1}{C_2} = \frac{\varepsilon \times 2 \times M_{CD}}{M_{PEG} + \varepsilon \times 2 \times M_{CD}}$, in which C_2 corresponds to the mass concentration of CD-PEG-CD, ε corresponds to the reaction efficiency, and M_{PEG} and M_{CD} correspond to the molecular weights of PEG and CD.

Determination of PEG-CD in the hydrogel network

For the determination of PEG-CD remaining in hydrogels, the hydrogel sample after gelation was immersed in ddH_2O for more than 12 hours. Then, the leachate was mixed with glycine-HCl buffer solution containing 1.2 mM methyl orange. Then, the concentration of PEG-CD in the mixture was determined using an ultraviolet visible (UV–vis) spectrophotometer according to the calibration curves. The ratio of PEG-CD being incorporated into the hydrogel network was calculated as (1- W_t/W_0) × 100%, in which W_t corresponds to the mass of CD in the mixture and W_0 corresponds to the total mass of CD added to the precursors.

For the determination of the CD ring filled by the PEG chain in hydrogels, the hydrogel sample after swelling was immersed in glycine-HCl buffer solution containing 1.2 mM methyl orange for more than 1 hour. Then, the concentration of CD that was not filled

with PEG in the hydrogel was determined using an ultraviolet visible (UV–vis) spectrophotometer according to the calibration curves. The ratio of the CD ring that has been filled by the PEG chain in hydrogels was calculated as (1-W_1/W_0) × 100%, in which W_1 corresponds to the mass of CD that remained unfilled with PEG in hydrogels and W_0 corresponds to the total mass of CD added to the precursors.

Swelling and water content measurements

In a typical swelling experiment, the volume of the initial hydrogel was recorded as V_1. Subsequently, the hydrogel was immersed in deionized water fiftyfold for 24 hours at room temperature to attain swelling equilibrium, and the volume was recorded as V_2. The swelling ratio was calculated as V_2/V_1.

For the water content evaluation, the hydrogels were first dialyzed in deionized water for another 24 hours. The hydrogel samples were weighed, and the wet weight was recorded as W_1. Then, the hydrogel samples were lyophilized, and the weight was recorded as W_2. The water content was calculated as $(1-W_2/W_1)\times 100\%$.

Scanning electron microscopy (SEM)

SEM images were obtained using a Quanta scanning electron microscope (Quanta 200, FEI) at 20 kV. The samples were lyophilized prior to the measurement. The meshes were marked with ImageJ and the sizes were measured according to the image scale.

Determination of the diffusion speed at zero pulling forces

Based on the Bell-Evans model, the diffusion rate of CD under force (k_F) on PEG chain can be described by $k_F = k_0 exp\left[\frac{F\Delta x}{k_B T}\right]$, where F is the friction force, k_0 is the diffusion frequency at zero force, T is the absolute temperature, k_B is the Boltzmann constant and Δx is the distance of the reaction length over which the force must be applied to reach the transition state. The diffusion rate of CD under force (k_F) can also be determined as $\frac{v}{L}$, in which v is the pulling speed and L is the length of PEG unit (~0.24 nm). Thus, the correlation between friction force (F) and pulling speed (v) in

SMFS follows the equation: $F = \frac{k_B T}{\Delta x} \ln \frac{v}{L k_0}$. By fitting the curves of F vs. v in Fig. 2d and e, the diffusion frequencies at zero force (k_0) were obtained. At last, the diffusion speeds of CD at zero pulling forces (D) were determined as $D = k_0 L$.

Rheological Measurements

The columned hydrogels (diameter ~ 8 mm, thickness ~ 3 mm) were carefully transferred to the rheometer plate of a rheometer (DHR-2, TA, USA) prior to the measurement. The rheology experiments at the frequency-sweep mode were carried out with a frequency of 0.01 rad s^{-1} to 100 rad s^{-1} at 1% strain (gap: 2.7 mm). The rheology experiments at the strain-sweep mode were carried out with a strain of 0.1 to 100 % at 1 Hz (gap: 2.7 mm). The rheological stress-relaxation experiments were performed using the stress relaxation mode at a strain amplitude of 20% (gap: 2.7 mm). The temperature was 25 °C for all rheological measurements.

Supplementary Figures

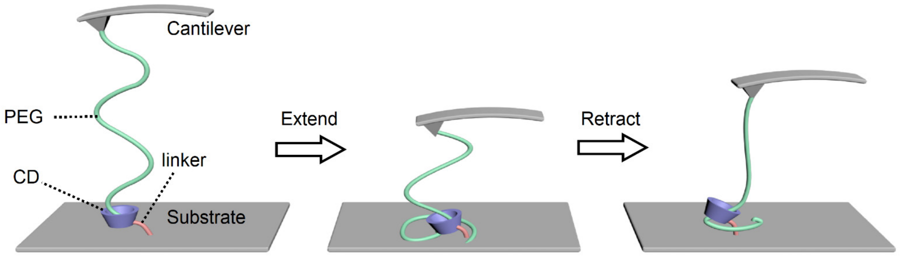

Supplementary Figure 1. Detailed schematic of the AFM-based SMFS experiments for the friction between PEG and CD. The PEG chain can thread through the CD ring when the cantilever is extended to the substrate. Subsequently, PEG was disengaged from the CD rings as the cantilever retracted from the substrate, and the friction between the PEG chain and CD was recorded during this process.

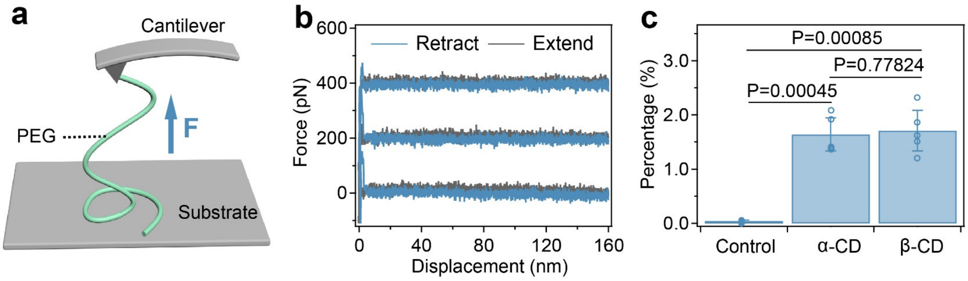

Supplementary Figure 2. SMFS on glass substrates without CD. **a,** Schematic diagram of the AFM-based SMFS experiments on glass substrates without CD. mPEG-SH (10 kDa) was linked to the cantilever tip via APTES and SMCC. **F** indicates the pulling force. **b,** Typical force–displacement curves of SMFS on glass substrates without CD at a pulling speed of 200 nm s^{-1}. In most traces, no force plateau was observed. **c,** Pickup rates (the success rate to obtain force-extension curves showing the force plateau) of SMFS on substrates modified with and without CD at a pulling speed of 200 nm s^{-1}. Values represent the mean and standard deviation (n = 5). *P* values, two-tailed Student's t test.

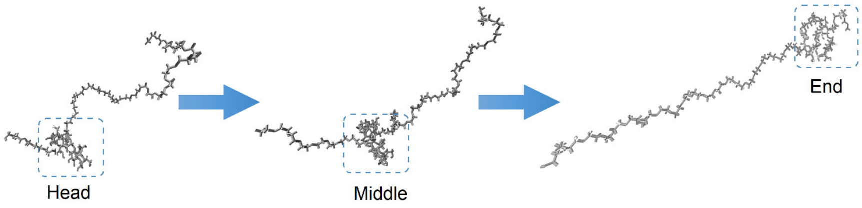

Supplementary Figure 3. Cartoon representations for different states of pulling a PEG (N = 34) through the ring of CD during a typical MD simulation. All the force fields of molecules were generated based on the general AMBER force field (GAFF) and Antechamber.

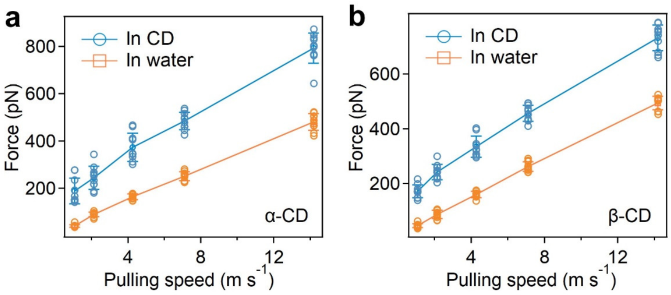

Supplementary Figure 4. Pulling forces of PEG in the CD ring and water at different pulling speeds in MD simulations. **a,** Pulling forces in the α-CD ring and water at different pulling speeds (1 to 14 m s^{-1}) in MD simulations. Values represent the mean and standard deviation (n = 10). **b,** Pulling forces in the β-CD ring and water at different pulling speeds (1 to 14 m s^{-1}) in MD simulations. Values represent the mean and standard deviation (n = 10).

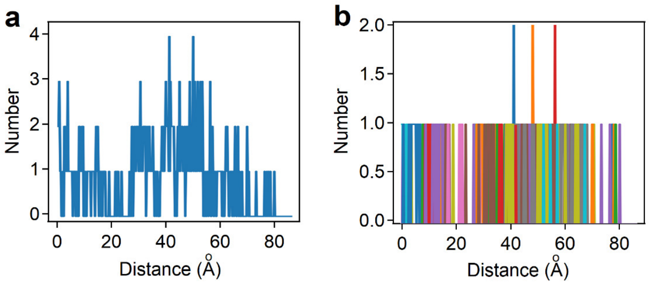

Supplementary Figure 5. Hydrogen bond in MD simulations for the sliding between PEG and α-CD. **a,** Fluctuation of the total hydrogen bond number with the pulling of PEG through the ring of α-CD. **b,** Formation and rupture of hydrogen bonds between a fixed oxygen atom of PEG and the α-CD ring.

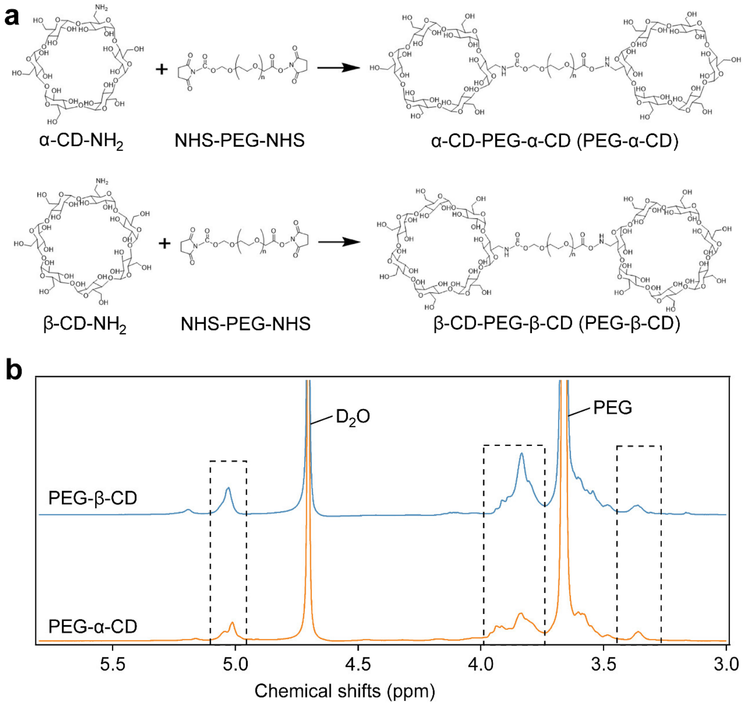

Supplementary Figure 6. Schematic of PEG-CD synthesis (**a**) and ^{1}H magnetic resonance spectroscopy (**b**) of PEG-α-CD and PEG-β-CD. The peaks highlighted by the dashed box correspond to α-CD and β-CD. The solvent used for the measurement was Deuterium oxide (D_2O).

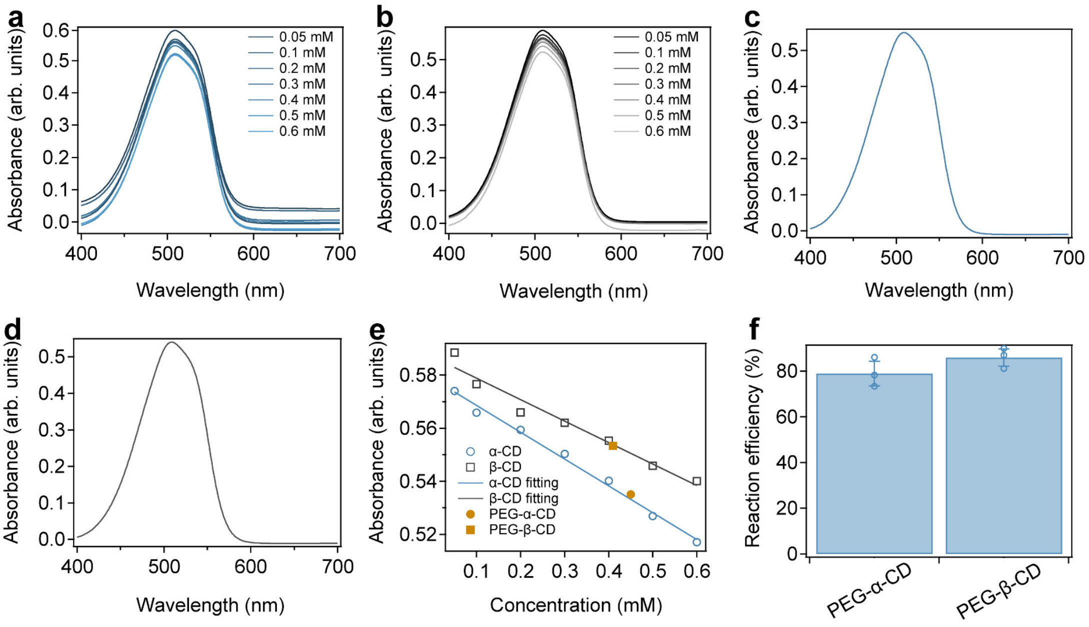

Supplementary Figure 7. Reaction efficiency of the synthesis of PEG-α-CD and PEG-β-CD. **a,** UV spectra of the mixture of α-CD and methyl orange (1.2 mM) at different

concentrations of α-CD (0.05-0.6 mM). **b,** UV spectra of the mixture of β-CD and methyl orange (1.2 mM) at different concentrations of β-CD (0.05-0.6 mM). **c,** UV spectra of the mixture of PEG-α-CD (1 mg mL^{-1}) and methyl orange (1.2 mM). **d,** UV spectra of the mixture of PEG-β-CD (1 mg mL^{-1}) and methyl orange (1.2 mM). **e,** Calibration curve of $OD_{509\ nm}$ and concentrations of α-CD and β-CD. The solid dots represent the concentrations and $OD_{509\ nm}$ for curves in **c** and **d**. **f,** Reaction efficiency for the synthesis of PEG-α-CD and PEG-β-CD. Values represent the mean and standard deviation (n = 3).

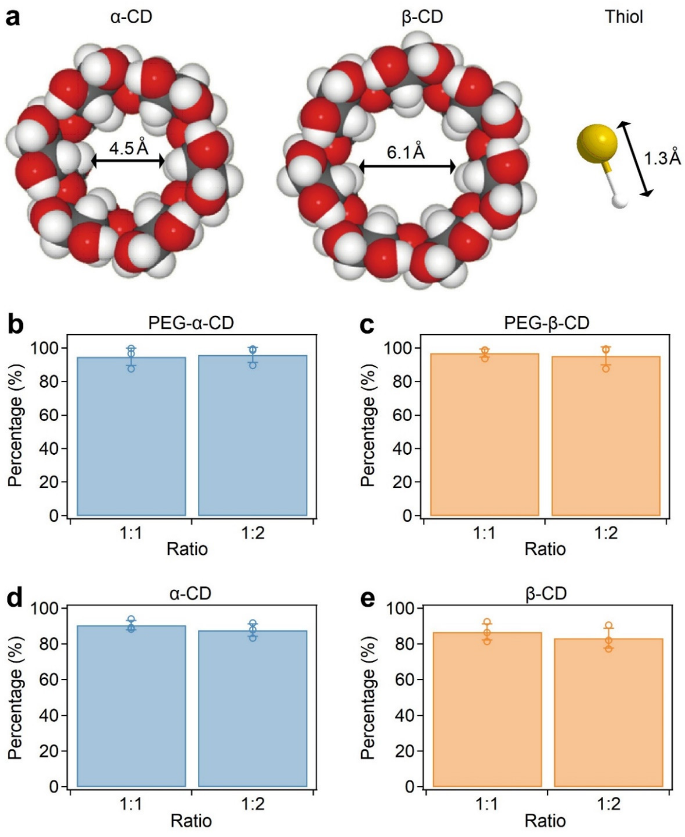

Supplementary Figure 8. Diffusion of PEG-CD and percentage of CD filled by the PEG chain in hydrogels. **a,** Cartoons of α-CD, β-CD and thiol. **b, c,** Percentages of PEG-α-CD (**b**) and PEG-β-CD (**c**) being incorporated into the hydrogel network determined by UV–vis spectroscopy. **d, e,** Percentages of α-CD (**d**) and β-CD (**e**), which have been filled by PEG chains in hydrogels. Values in **b**-**e** represent the mean and

standard deviation (n = 3).

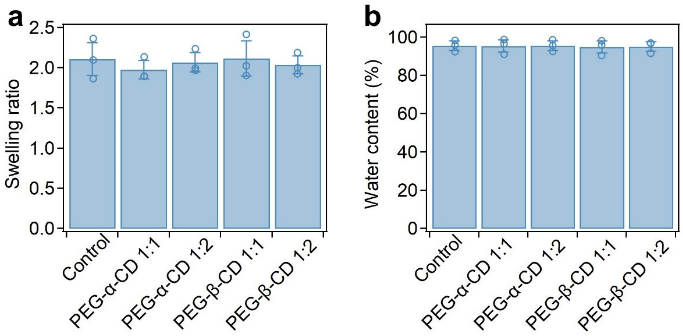

Supplementary Figure 9. Swelling ratios (**a**) and water contents (**b**) of hydrogels at different PEG-SH:PEG-CD ratios (1:1 and 1:2). The hydrogel prepared without chain walkers was used as a control. Values represent the mean and standard deviation (n = 3).

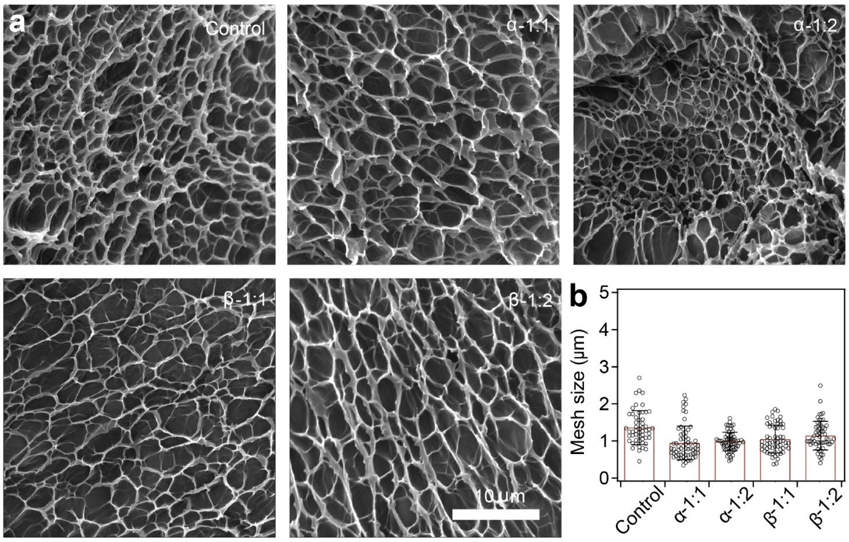

Supplementary Figure 10. SEM images and mesh sizes of different hydrogels. **a,** SEM images of hydrogels at different PEG-SH:PEG-CD ratios (1:1 and 1:2). The hydrogel prepared without PEG-CD was used as a control. **b,** Average mesh sizes of different hydrogels. Values represent the mean and standard deviation (n>50). Each experiment was repeated 3 times independently with similar results.

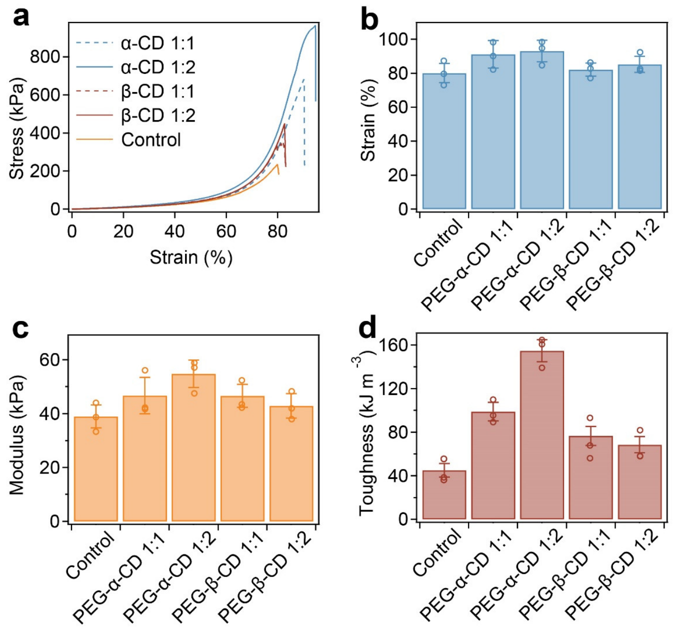

Supplementary Figure 11. Mechanical properties of hydrogels containing different chain walkers. **a,** Stress–strain curves of different hydrogels under compression. **b,** Fracture strain of different hydrogels under compression. **c,** Young's modulus of different hydrogels under compression. **d,** Toughness of different hydrogels under compression. Values in **b-d** represent the mean and standard deviation (n = 3).

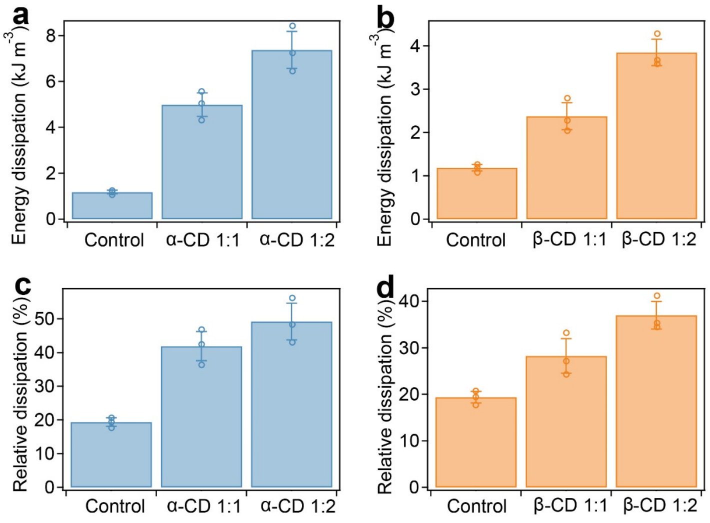

Supplementary Figure 12. Energy dissipation and relative energy dissipation of different hydrogels. **a, b,** Energy dissipation of hydrogels containing PEG-α-CD (**a**) or PEG-β-CD (**b**) at different ratios of PEG-SH and PEG-CD. **c, d,** Relative energy

dissipation of hydrogels containing PEG-α-CD (**c**) or PEG-β-CD (**d**) at different ratios of PEG-SH and PEG-CD. Values represent the mean and standard deviation (n = 3).

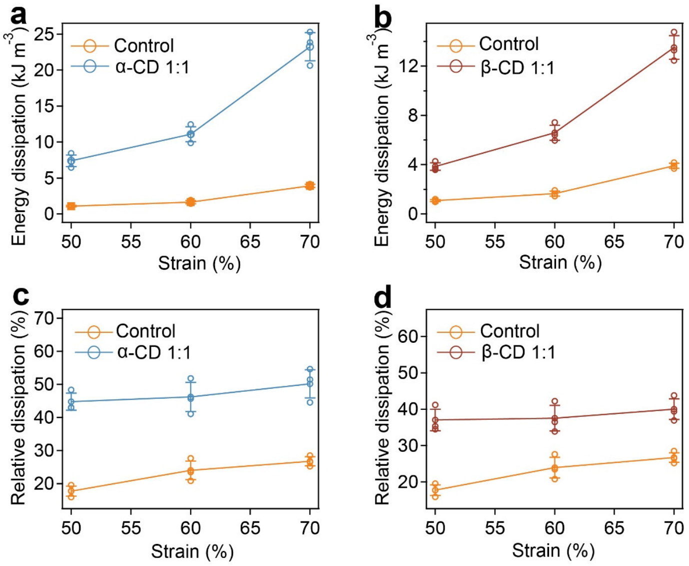

Supplementary Figure 13. Energy dissipation and relative energy dissipation of different hydrogels under different strains. **a, b,** Energy dissipation of hydrogels containing PEG-α-CD (**a**) or PEG-β-CD (**b**) under different strains (50, 60 and 70%) at a PEG-SH:PEG-CD ratio of 1:1. **c, d,** Relative energy dissipation of hydrogels containing PEG-α-CD (**c**) or PEG-β-CD (**d**) under different strains (50, 60 and 70%) at a PEG-SH:PEG-CD ratio of 1:1. Values represent the mean and standard deviation (n = 3).

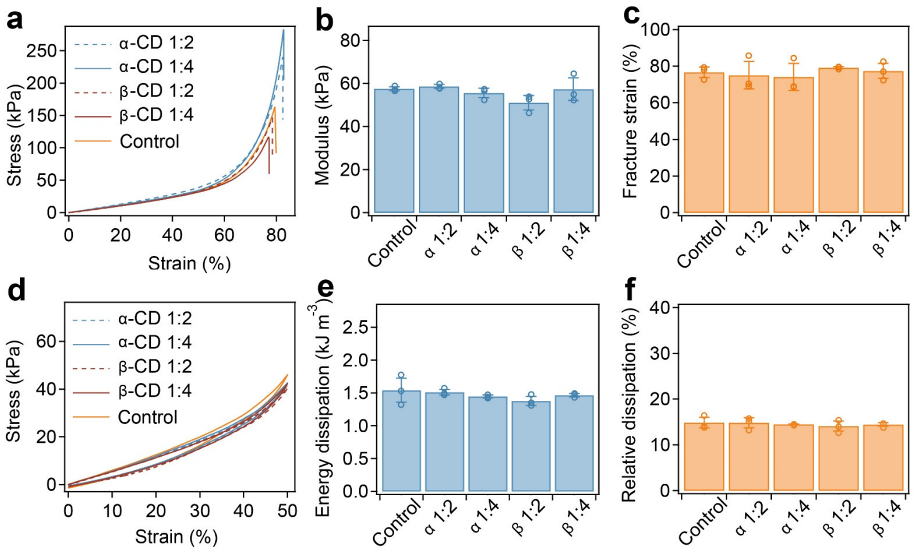

Supplementary Figure 14. Mechanical properties of hydrogels prepared with CD monomers that were not linked via the PEG segment. The concentrations of CD monomers used in the preparation were twice those of CD-PEG-CD, as each CD-PEG-CD (chain walker) contains two CD rings. Hydrogels without CD were used as control. **a,** Typical stress–strain curves of hydrogels at different PEG-SH:CD ratios (1:2 and 1:4). **b, c,** Summarized moduli (**b**) and fracture strains (**c**) of hydrogels at different PEG-SH:CD ratios (1:2 and 1:4). Values represent the mean and standard deviation (n = 3). **d,** Typical compression-relaxation curves of hydrogels at different PEG-SH:CD ratios (1:2 and 1:4) at a strain of 50%. **e, f,** Summarized energy dissipation (**e**) and relative energy dissipation (**f**) of hydrogels at different PEG-SH:CD ratios (1:2 and 1:4). Values represent the mean and standard deviation (n = 3).

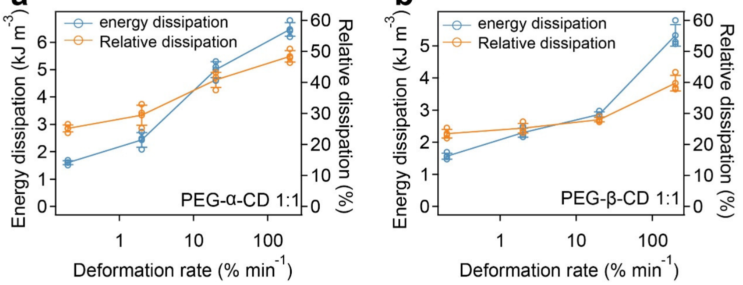

Supplementary Figure 15. Energy dissipation and relative energy dissipation of hydrogels containing PEG-CD at various deformation rates. **a,** Energy dissipation and

relative energy dissipation of hydrogels containing PEG-α-CD at various deformation rates (0.2%, 2%, 20%, and 200% min^{-1}) at a PEG-SH:PEG-α-CD ratio of 1:1. **b,** Energy dissipation and relative energy dissipation of hydrogels containing PEG-β-CD at various deformation rates (0.2%, 2%, 20%, and 200% min^{-1}) at a PEG-SH:PEG-β-CD ratio of 1:1. Values represent the mean and standard deviation (n = 3).

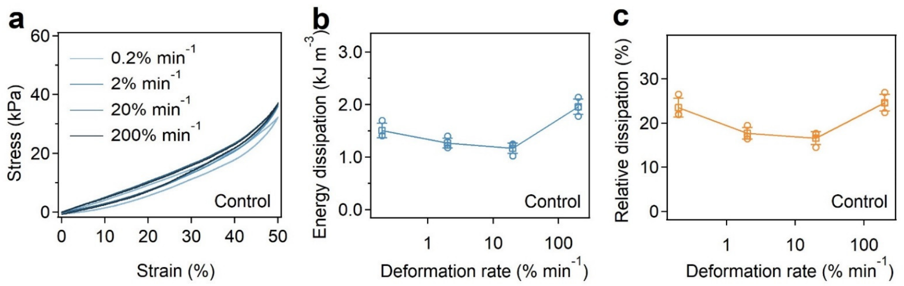

Supplementary Figure 16. Rate-dependent performance of hydrogels without PEG-CD. **a**, Compress-relaxation curves of hydrogels without PEG-CD at different deformation rates (0.2%, 2%, 20% and 200% min^{-1}) at a strain of 50%. **b**, **c**, Energy dissipation and relative energy dissipation of hydrogels without PEG-CD at different deformation rates (0.2%, 2%, 20% and 200% min^{-1}) at a strain of 50%. Values represent the mean and standard deviation (n = 3).

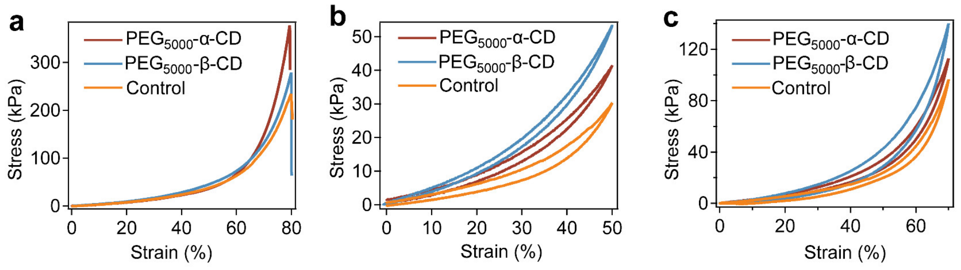

Supplementary Figure 17. Mechanical properties of hydrogels containing PEG$_{5000}$-CD. PEG$_{5000}$-CD refers to α-CD or β-CD linked with PEG at a molecular weight of 5 kDa. **a,** Stress–strain curves of hydrogels containing PEG$_{5000}$-α-CD or PEG$_{5000}$-β-CD under compression (PEG-SH: PEG$_{5000}$-CD = 1:1). **b, c,** Typical compression-relaxation curves of hydrogels containing PEG$_{5000}$-α-CD or PEG$_{5000}$-β-CD at strains of 50% (**b**)

and 70% (**c**). The molar ratio of PEG-SH and PEG_{5000}-CD was 1:1. Each experiment was repeated 3 times independently with similar results.

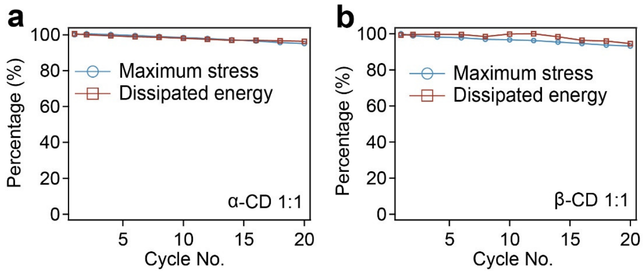

Supplementary Figure 18. Summarized maximum stress and energy dissipation of hydrogels containing PEG-α-CD (**a**) or PEG-β-CD (**b**) at a PEG-SH:PEG-CD ratio of 1:1 in 20 cycles of continuous compression-relaxation.

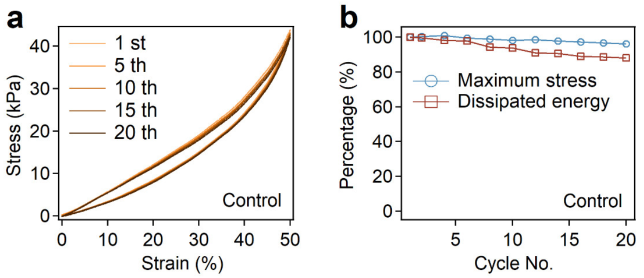

Supplementary Figure 19. Recovery performance of hydrogels without PEG-CD under consecutive compression-relaxation cycles. **a**, Consecutive compression-relaxation cycles of hydrogels without PEG-CD for 20 cycles at the deformation rate of 20% min^{-1} without any waiting time. **b**, Normalized maximum stress and dissipated energy of hydrogels without PEG-CD in 20 cycles of continuous compression-relaxation.

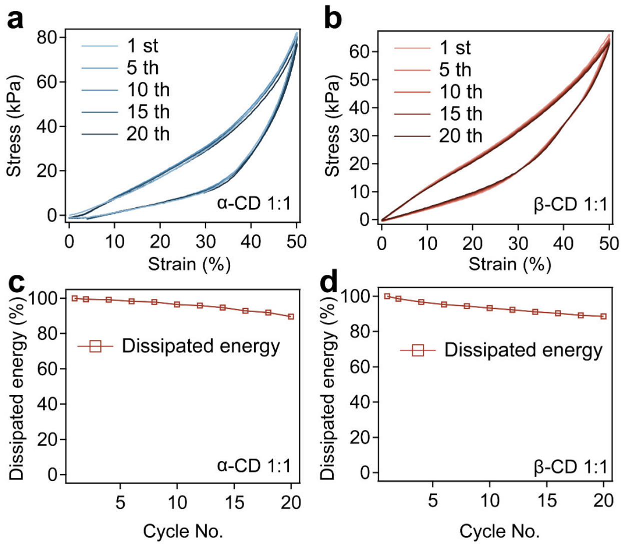

Supplementary Figure 20. Energy dissipation of damping hydrogels at high deformation rates. **a,** Consecutive compression-relaxation cycles of hydrogels containing PEG-α-CD without any waiting time for 20 cycles at the deformation rate of 200% min^{-1} (PEG-SH:PEG-α-CD = 1:1). **b,** Consecutive compression-relaxation cycles of hydrogels containing PEG-β-CD without any waiting time for 20 cycles at the deformation rate of 200% min^{-1} (PEG-SH:PEG-β-CD = 1:1). **c, d,** Normalized energy dissipation of hydrogels containing PEG-α-CD (**c**) or PEG-β-CD (**d**) in 20 consecutive compression-relaxation cycles at the deformation rate of 200% min^{-1} (PEG-SH:PEG - CD = 1:1).

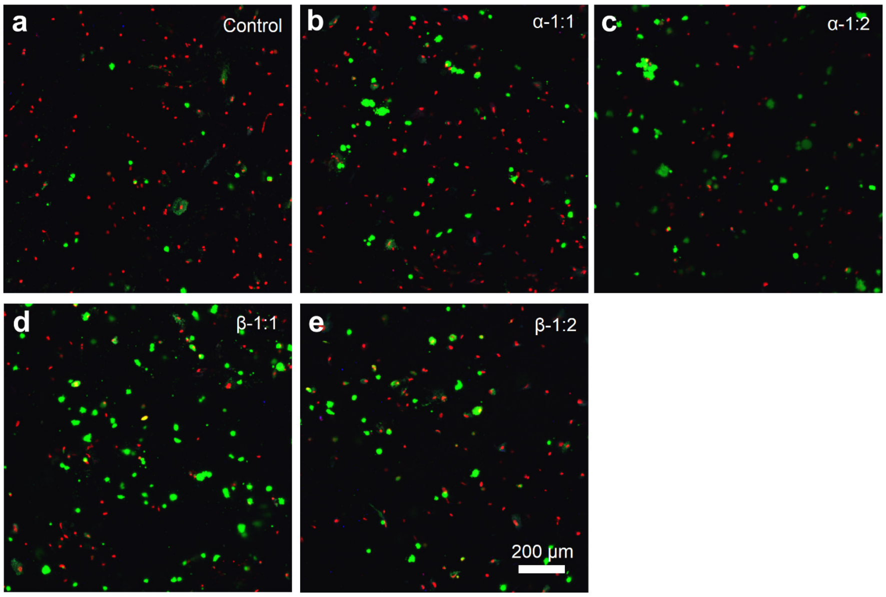

Supplementary Figure 21. Typical projected images of 3D reconstructions in the Z-

axis direction for live/dead cell staining in hydrogels after 2000 cycles of compression-relaxation (strain ~ 60%). **a,** Hydrogel without PEG-CD. **b, c,** Hydrogel at PEG-SH:PEG-α-CD ratios of 1:1 (**b**) and 1:2 (**c**). **d, e,** Hydrogel at PEG-SH:PEG-β-CD ratios of 1:1 (**d**) and 1:2 (**e**). Each experiment was repeated 5 times independently with similar results.

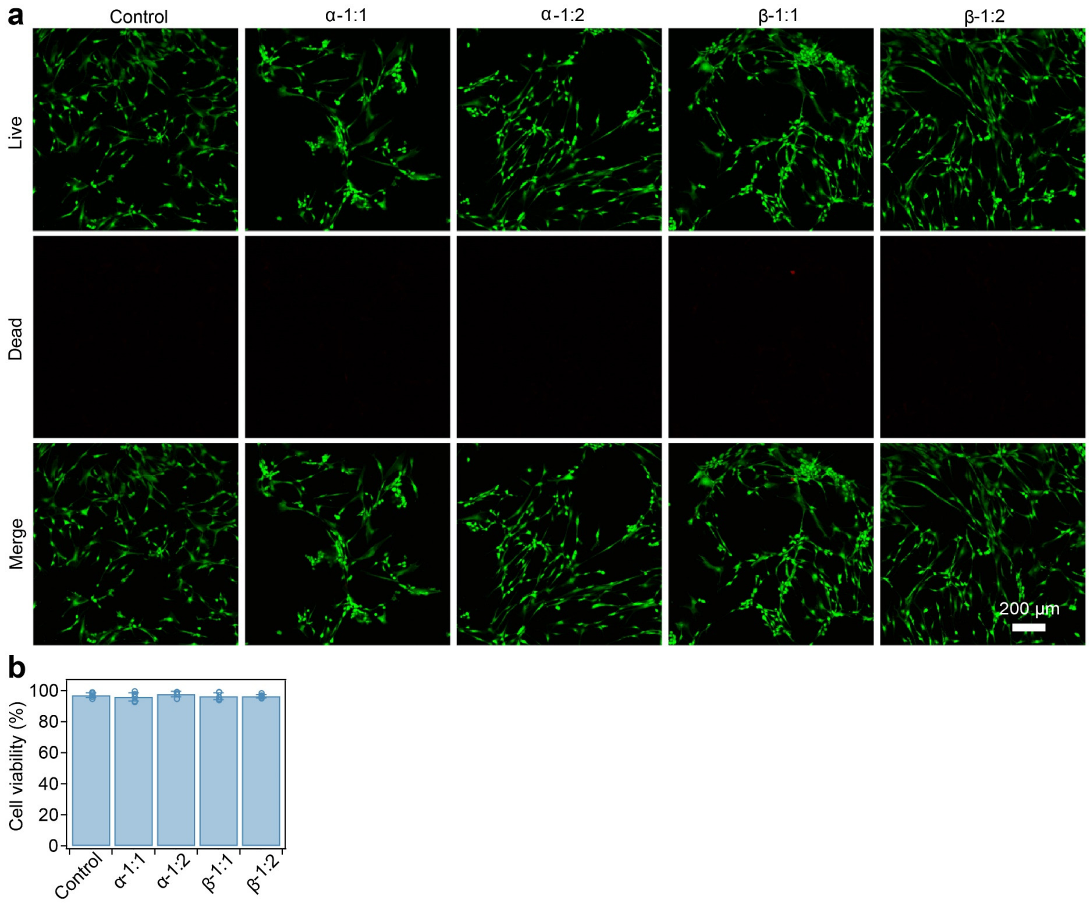

Supplementary Figure 22. Cell viabilities of hMSCs cultured on different hydrogels for 24 hours. Cells cultured on cell culture plates were used as control groups. **a,** Live/dead staining of hMSCs on different hydrogels after being cultured for 24 hours. Green represents live cells with high enzymatic activity indicated by calcein-AM. The red color of PI shows dead cells with compromised membranes. Each experiment was repeated 5 times independently with similar results. **b,** Viabilities determined by live/dead staining of hMSCs on different hydrogels after culture for 24 hours. Values represent the mean and the standard deviation (n = 5).

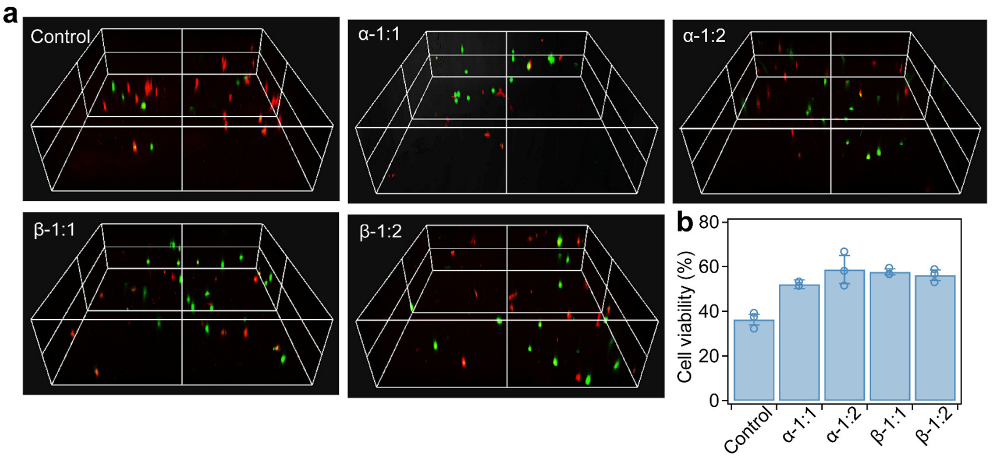

Supplementary Figure 23. Cell shielding in hydrogels ($C_{PEG\text{-}Mal} = C_{PEG\text{-}SH} = 70$ mg mL^{-1}) in the presence and absence of PEG-CD after 2000 cycles of compression-relaxation (strain ~ 30%). **a,** 3D reconstructions of live/dead cell staining in different hydrogels using laser confocal fluorescence microscopy (LCFM) after 2000 cycles of compression-relaxation (strain ~ 30%). Cells were stained using calcein-AM (green) and propidium iodide (PI) (red). The size of the scanning space was 1272 μm × 1272 μm × 300 μm. Each experiment was repeated 3 times independently with similar results. **b,** Cell viabilities of hMSCs in different hydrogels after 2000 cycles of compression-relaxation. Values represent the mean and standard deviation (n = 3).

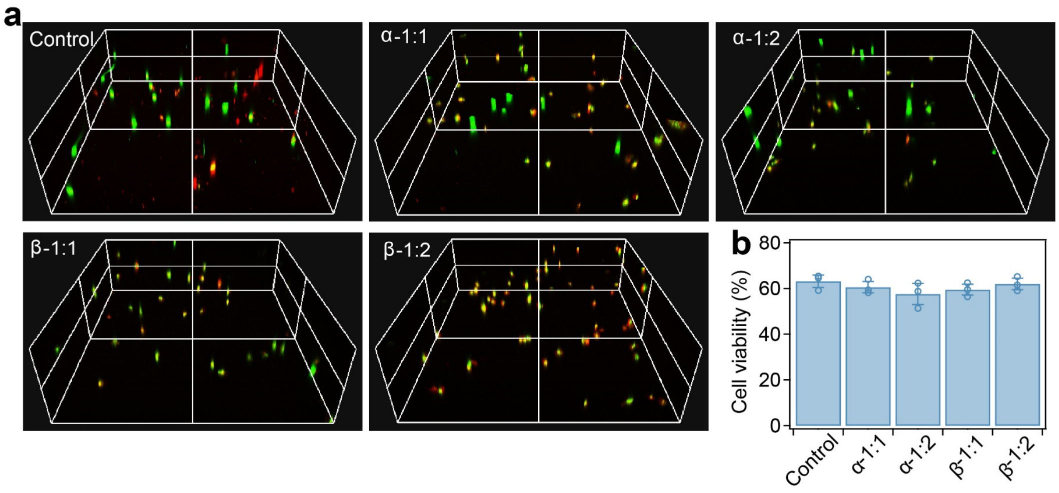

Supplementary Figure 24. Live/dead cell staining in hydrogels ($C_{PEG\text{-}Mal} = C_{PEG\text{-}SH} =$

70 mg mL^{-1}) in the presence and absence of PEG-CD without undergoing cyclic compression-relaxation. **a,** 3D reconstructions of live/dead cell staining in different hydrogels using laser confocal fluorescence microscopy (LCFM). Cells were stained using calcein-AM (green) and propidium iodide (PI) (red). The size of the scanning space was 1272 μm × 1272 μm × 300 μm. Each experiment was repeated 3 times independently with similar results. **b,** Cell viabilities of hMSCs in different hydrogels after 2000 cycles of compression-relaxation. Values represent the mean and standard deviation (n = 3).

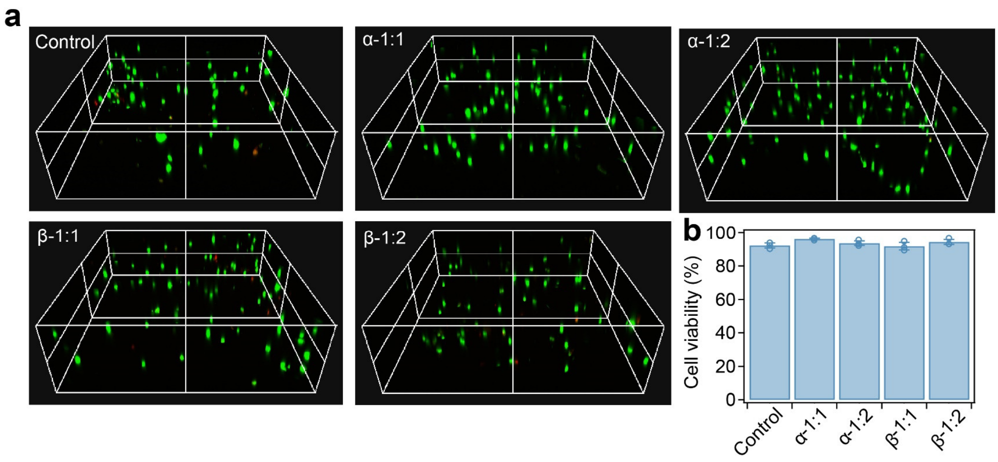

Supplementary Figure 25. Live/dead cell staining in hydrogels ($C_{PEG-Mal} = C_{PEG-SH} =$ 20 mg mL^{-1}) in the presence and absence of PEG-CD without undergoing cyclic compression-relaxation. **a,** 3D reconstructions of live/dead cell staining in different hydrogels using laser confocal fluorescence microscopy (LCFM). Cells were stained using calcein-AM (green) and propidium iodide (PI) (red). The size of the scanning space was 1272 μm × 1272 μm × 300 μm. Each experiment was repeated 3 times independently with similar results. **b,** Cell viabilities of hMSCs in different hydrogels. Values represent the mean and standard deviation (n = 3).

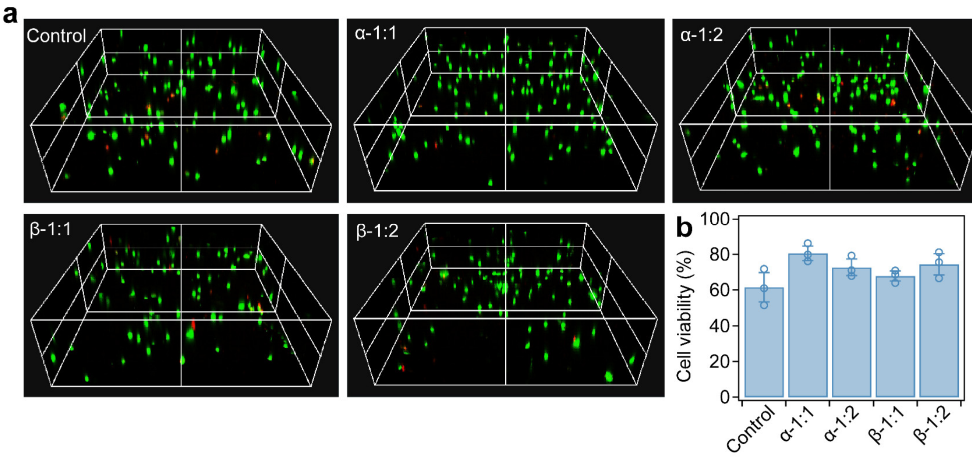

Supplementary Figure 26. Cell shielding in hydrogels ($C_{PEG\text{-}Mal} = C_{PEG\text{-}SH} = 20$ mg mL^{-1}) in the presence and absence of PEG-CD after 2000 cycles of compression-relaxation (strain: 30%). **a,** 3D reconstructions of live/dead cell staining in different hydrogels using laser confocal fluorescence microscopy (LCFM) after 2000 cycles of compression-relaxation (strain: 30%). Cells were stained using calcein-AM (green) and propidium iodide (PI) (red). The size of the scanning space was 1272 μm × 1272 μm × 300 μm. Each experiment was repeated 3 times independently with similar results. **b,** Cell viabilities of hMSCs in different hydrogels after 2000 cycles of compression-relaxation (strain: 30%). Values represent the mean and standard deviation (n = 3).

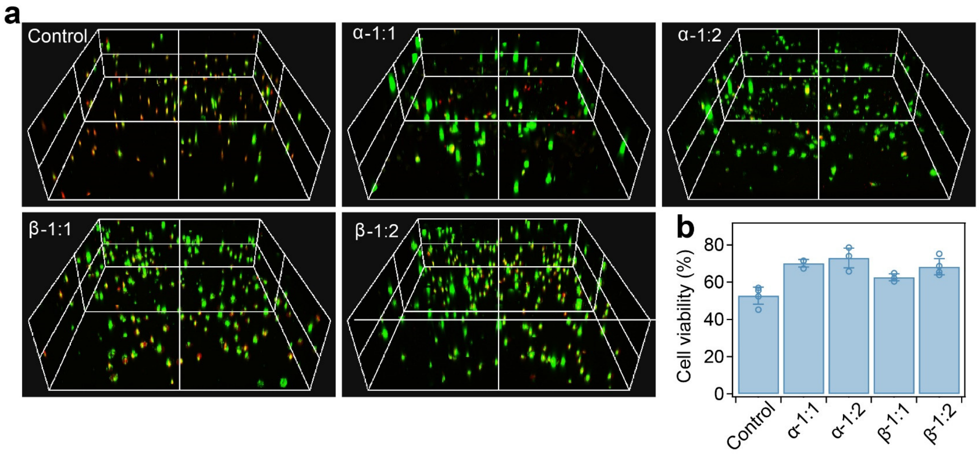

Supplementary Figure 27. Cell shielding in hydrogels ($C_{PEG\text{-}Mal} = C_{PEG\text{-}SH} = 20$ mg mL^{-1}) in the presence and absence of PEG-CD after 2000 cycles of compression-relaxation (strain: 60%). **a,** 3D reconstructions of live/dead cell staining in different

hydrogels using laser confocal fluorescence microscopy (LCFM) after 2000 cycles of compression-relaxation (strain: 60%). Cells were stained using calcein-AM (green) and propidium iodide (PI) (red). The size of the scanning space was 1272 μm × 1272 μm × 300 μm. Each experiment was repeated 3 times independently with similar results. **b,** Cell viabilities of hMSCs in different hydrogels after 2000 cycles of compression-relaxation (strain: 60%). Values represent the mean and standard deviation (n = 3-4).

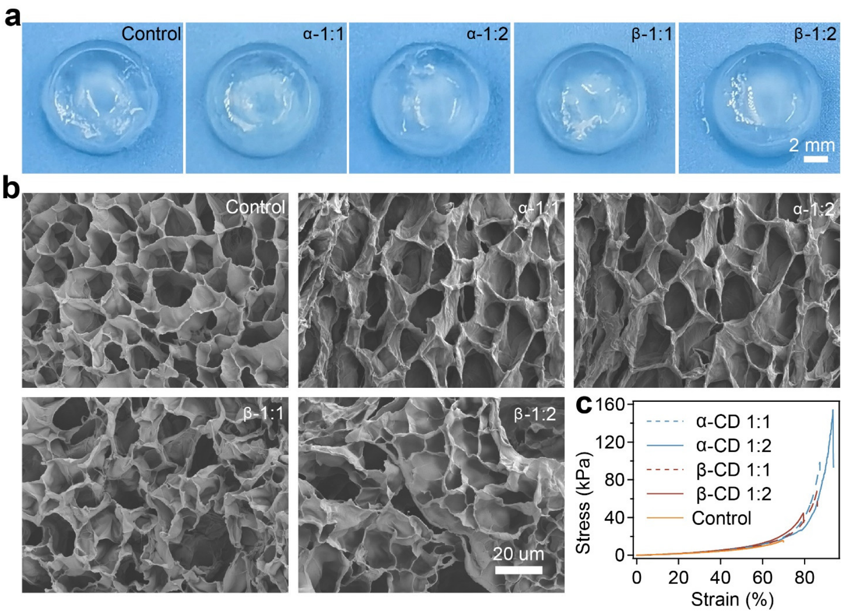

Supplementary Figure 28. Characterization of hydrogels at low PEG concentrations ($C_{PEG\text{-}Mal} = C_{PEG\text{-}SH} = 20$ mg mL^{-1}). **a, b,** Optical (**a**) and SEM (**b**) images of different hydrogels at low PEG concentrations ($C_{PEG\text{-}Mal} = C_{PEG\text{-}SH} = 20$ mg mL^{-1}). **c,** Stress–strain curves of different hydrogels under compression at low PEG concentrations ($C_{PEG\text{-}Mal} = C_{PEG\text{-}SH} = 20$ mg mL^{-1}). Each experiment was repeated 3 times independently with similar results.

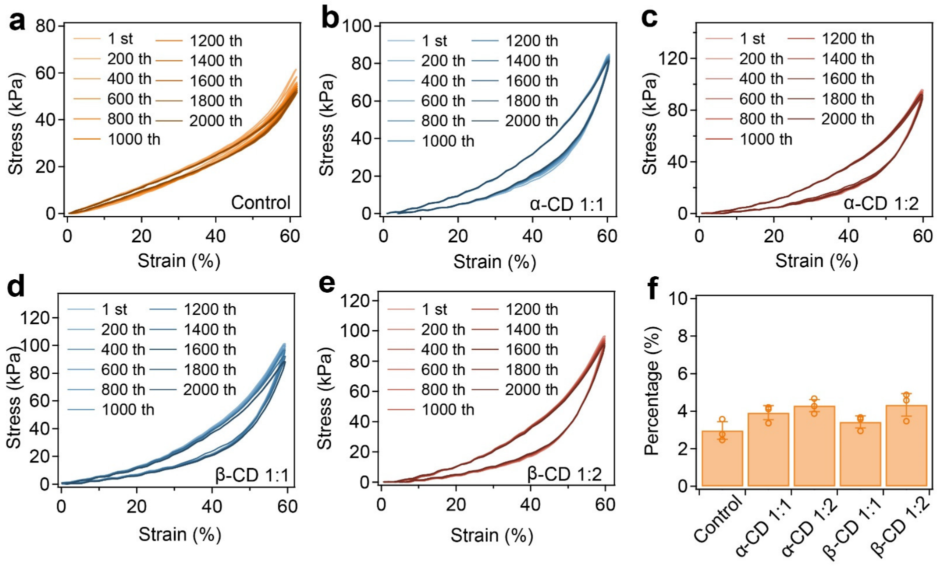

Supplementary Figure 29. Mechanical performances of different hydrogels in 2000 cycles of compression-relaxation. **a,** Consecutive compression-relaxation cycles of control hydrogels without any waiting time for 2000 cycles (frequency ~0.5 Hz). **b,** Consecutive compression-relaxation cycles of hydrogels containing PEG-α-CD without any waiting time for 2000 cycles (PEG-SH:PEG-α-CD = 1:1, frequency ~0.5 Hz). **c,** Consecutive compression-relaxation cycles of hydrogels containing PEG-α-CD without any waiting time for 2000 cycles (PEG-SH:PEG-α-CD = 1:2, frequency ~0.5 Hz). **d,** Consecutive compression-relaxation cycles of hydrogels containing PEG-β-CD without any waiting time for 2000 cycles (PEG-SH:PEG-β-CD = 1:1, frequency ~0.5 Hz). **e,** Consecutive compression-relaxation cycles of hydrogels containing PEG-β-CD without any waiting time for 2000 cycles (PEG-SH:PEG-β-CD = 1:2, frequency ~0.5 Hz). **f,** Summarized permanent deformations of different hydrogels after 2000 cycles of compression-relaxation. Values represent the mean and standard deviation (n = 3).

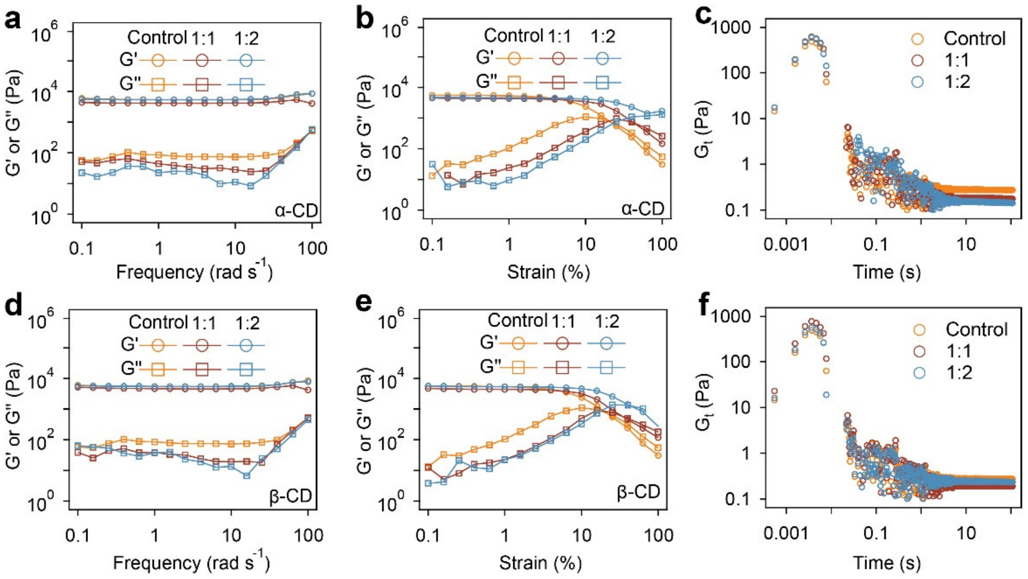

Supplementary Figure 30. Rheological characterization of different hydrogels. The hydrogel prepared without chain walkers was used as the control. **a**, **b**, Rheological measurements of hydrogels at different PEG-SH:PEG-α-CD ratios (1:1 and 1:2) under the frequency-sweep (**a**) and strain-sweep (**b**) modes. **c**, Stress relaxation analysis for hydrogels at different PEG-SH:PEG-α-CD ratios (1:1 and 1:2) based on rheology measurements. **d**, **e**, Rheological measurements of hydrogels at different PEG-SH:PEG-β-CD ratios (1:1 and 1:2) under the frequency-sweep (**d**) and strain-sweep (**e**) modes. **f**, Stress relaxation analysis for hydrogels at different PEG-SH:PEG-β-CD ratios (1:1 and 1:2) based on rheology measurements. For **c** and **f**, the rapid stress relaxation observed within the initial second is likely due to artifacts caused by the overshooting of the test probe during engagement with the hydrogels. This overshooting led to mechanical perturbations, resulting in signals that do not reflect the true stress relaxation of the hydrogel network. Only the stress relaxation profiles (*G(t)*) measured after this initial phase are reliable, consistently showing no significant relaxation for all three hydrogels tested within the 1-100 second timeframe.

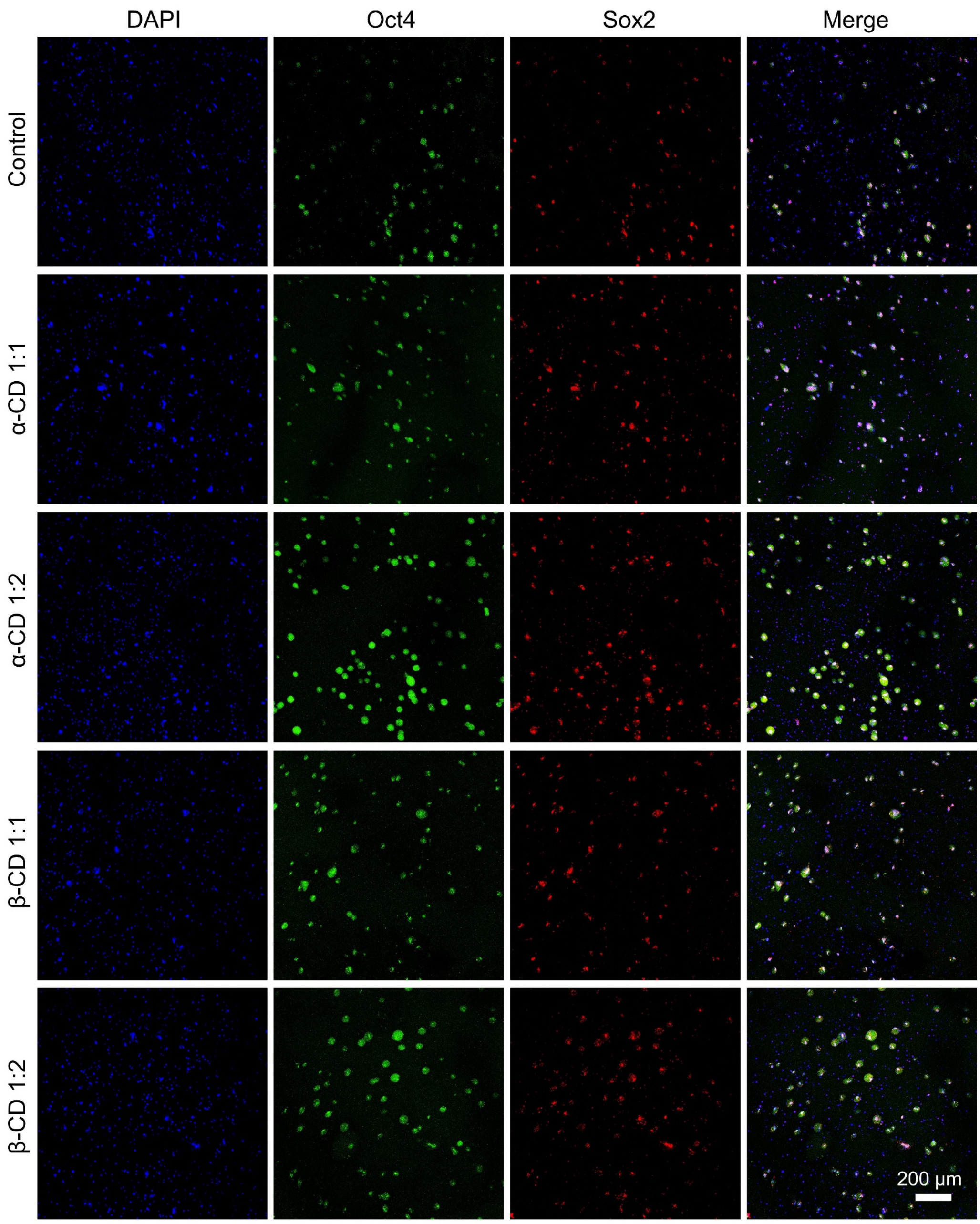

Supplementary Figure 31. Stemness of hMSCs in hydrogels ($C_{PEG\text{-}Mal} = C_{PEG\text{-}SH} = 70$ mg mL^{-1}) after 2000 cycles of compression-relaxation (strain ~ 60%). A space of 1272 μm × 1272 μm × 300 μm was scanned, and the projected image in the Z-axis direction is shown. Immunofluorescence staining of specific markers for stemness maintenance, Oct4 (green) and Sox2 (red), was used to evaluate the stemness of hMSCs. Cell nuclei are indicated by DAPI (blue). Each experiment was repeated 5 times independently with similar results.
