# Supplementary material for: Rapidly damping hydrogels engineered through molecular friction
Source: Nat Commun. 2024 Jun 8;15:4895. doi: 10.1038/s41467-024-49239-4 (PMC11162443; doi:10.1038/s41467-024-49239-4)
Supplement: Supplementary file 3 — Reporting Summary [file 41467_2024_49239_MOESM3_ESM.pdf]

nature portfolio

Corresponding author(s): Yi Cao

Last updated by author(s): May 26, 2024

Reporting Summary

Nature Portfolio wishes to improve the reproducibility of the work that we publish. This form provides structure for consistency and transparency in reporting. For further information on Nature Portfolio policies, see our Editorial Policies and the Editorial Policy Checklist.

Statistics

For all statistical analyses, confirm that the following items are present in the figure legend, table legend, main text, or Methods section.

n/a	Confirmed	
☐	☒	The exact sample size (*n*) for each experimental group/condition, given as a discrete number and unit of measurement
☐	☒	A statement on whether measurements were taken from distinct samples or whether the same sample was measured repeatedly
☐	☒	The statistical test(s) used AND whether they are one- or two-sided<br>*Only common tests should be described solely by name; describe more complex techniques in the Methods section.*
☒	☐	A description of all covariates tested
☒	☐	A description of any assumptions or corrections, such as tests of normality and adjustment for multiple comparisons
☐	☒	A full description of the statistical parameters including central tendency (e.g. means) or other basic estimates (e.g. regression coefficient) AND variation (e.g. standard deviation) or associated estimates of uncertainty (e.g. confidence intervals)
☐	☒	For null hypothesis testing, the test statistic (e.g. *F*, *t*, *r*) with confidence intervals, effect sizes, degrees of freedom and *P* value noted<br>*Give P values as exact values whenever suitable.*
☒	☐	For Bayesian analysis, information on the choice of priors and Markov chain Monte Carlo settings
☒	☐	For hierarchical and complex designs, identification of the appropriate level for tests and full reporting of outcomes
☒	☐	Estimates of effect sizes (e.g. Cohen's *d*, Pearson's *r*), indicating how they were calculated

Our web collection on statistics for biologists contains articles on many of the points above.

Software and code

Policy information about availability of computer code

Data collection	Data were collected with various laboratory instruments and equipments. Such as: tensile-compressive tester (Instron-5944, USA), atomic force microscope (JPK Nanowazrid II, Germany), ultraviolet visible (UV–vis) spectrophotometer (JASCO V550, Japan), Quanta scanning electron microscope (Quata 200, FEI, USA), and laser confocal fluorescence microscope (Olympus FV3000, Japan).
Data analysis	Igor6.37, JPK data processing software (7.0.46), ImageJ V 1.8.0, IBM SPSS statistics 26, Microsoft Excel 2019, VMD 1.9.4..

For manuscripts utilizing custom algorithms or software that are central to the research but not yet described in published literature, software must be made available to editors and reviewers. We strongly encourage code deposition in a community repository (e.g. GitHub). See the Nature Portfolio guidelines for submitting code & software for further information.

Data

Policy information about availability of data

All manuscripts must include a data availability statement. This statement should provide the following information, where applicable:

- Accession codes, unique identifiers, or web links for publicly available datasets
- A description of any restrictions on data availability
- For clinical datasets or third party data, please ensure that the statement adheres to our policy

All data are available in the main text or the Supplementary Information. The source data underlying all figures are provided as Source Data file.

Research involving human participants, their data, or biological material

Policy information about studies with human participants or human data. See also policy information about sex, gender (identity/presentation), and sexual orientation and race, ethnicity and racism.

Reporting on sex and gender	This work did not involve human research.
Reporting on race, ethnicity, or other socially relevant groupings	This work did not involve human research.
Population characteristics	This work did not involve human research.
Recruitment	This work did not involve human research.
Ethics oversight	This work did not involve human research.

Note that full information on the approval of the study protocol must also be provided in the manuscript.

Field-specific reporting

Please select the one below that is the best fit for your research. If you are not sure, read the appropriate sections before making your selection.

☒ Life sciences ☐ Behavioural & social sciences ☐ Ecological, evolutionary & environmental sciences

For a reference copy of the document with all sections, see nature.com/documents/nr-reporting-summary-flat.pdf

Life sciences study design

All studies must disclose on these points even when the disclosure is negative.

Sample size	Sample sizes were chosen based on previously published literature and protocols in the field with a similar setup that showed statistical significance. For each experiment, we have adopted at least n=3 biological replicates to calculate the statistical value of each analysis and the exact sample sizes and statistical data for each experiment are reported in the figure legends.
Data exclusions	No data were excluded from the analyses.
Replication	All attempts at replication were successful and the exact numbers are indicated in the figure legends or table for all experiments.
Randomization	All the samples were randomly grouped.
Blinding	Investigators are blinded to the group allocation during the data collection and analyses.

Reporting for specific materials, systems and methods

We require information from authors about some types of materials, experimental systems and methods used in many studies. Here, indicate whether each material, system or method listed is relevant to your study. If you are not sure if a list item applies to your research, read the appropriate section before selecting a response.

Materials & experimental systems

n/a	Involved in the study	
☐	☒	Antibodies
☐	☒	Eukaryotic cell lines
☒	☐	Palaeontology and archaeology
☒	☐	Animals and other organisms
☒	☐	Clinical data
☒	☐	Dual use research of concern
☒	☐	Plants

Methods

n/a	Involved in the study	
☒	☐	ChIP-seq
☒	☐	Flow cytometry
☒	☐	MRI-based neuroimaging

Antibodies

Antibodies used	The antibodies used in this study include anti-Sox2 antibody (cat: D9B8N, CST, USA), anti-Oct4 antibody (cat: D7O5Z, CST, USA), Alexa Fluor 647-labelled goat anti-rabbit IgG (H+L, cat: A0468, Beyotime, China) and FITC-labelled goat anti-mouse IgG (H+L, cat: E031210,

	Earthox, USA).
Validation	Anti-Sox2 antibody (cat: D9B8N, CST, USA, diluted 1:200), Rabbit, IF, https://www.cellsignal.cn/products/primary-antibodies/sox2-d9b8n-rabbit-mab/23064. Anti-Oct4 antibody (cat: D705Z, CST, USA, diluted 1:200), mouse, IF, https://www.cellsignal.cn/products/primary-antibodies/oct-4-d7o5z-mouse-mab/75463. Alexa Fluor 647-labelled goat anti-rabbit IgG (H+L, cat: A0468, Beyotime, China, diluted 1:200), https://m.beyotime.com/product/A0468.htm. FITC-labelled goat anti-mouse IgG (H+L, cat: E031210, Earthox, USA, diluted 1:200), https://earthox.net/product/fitc-goat-anti-mouse-igghl-e031210-02/.

Eukaryotic cell lines

Policy information about cell lines and Sex and Gender in Research

Cell line source(s)	Bone Marrow-Derived Mesenchymal Stem Cells; Normal, Human (ATCC® PCS-500-012™)
Authentication	https://www.atcc.org/en/products/cells_and_microorganisms/stem_cells/human_mesenchymal_stem_cells/pcs-500-012. The cells are authenticated by STR profiling.
Mycoplasma contamination	Bone marrow mesenchymal stem cells were tested negative for mycoplasma contamination.
Commonly misidentified lines (See ICLAC register)	None.

Plants

Seed stocks	This work did not involve plants.
Novel plant genotypes	This work did not involve plants.
Authentication	This work did not involve plants.
